# Plant-Derived Natural Biomolecule Picein Attenuates Menadione Induced Oxidative Stress on Neuroblastoma Cell Mitochondria

**DOI:** 10.3390/antiox9060552

**Published:** 2020-06-25

**Authors:** Kavindra Kumar Kesari, Anupam Dhasmana, Shruti Shandilya, Neeraj Prabhakar, Ahmed Shaukat, Jinze Dou, Jessica M. Rosenholm, Tapani Vuorinen, Janne Ruokolainen

**Affiliations:** 1 Department of Applied Physics, Aalto University, 00076 Espoo, Finland; shruti.shandilya@aalto.fi; 2 Department of Microbiology and Immunology, School of Medicine, University of Texas Rio Grande Valley, McAllen, TX 78539, USA; anudhas007@gmail.com; 3 Department of Biosciences, Swami Rama Himalayan University, Dehradun 248016, India; 4 Pharmaceutical Sciences Laboratory, Faculty of Science and Engineering, Åbo Akademi University, 20500 Turku, Finland; Neeraj.Prabhakar@abo.fi (N.P.); jessica.rosenholm@abo.fi (J.M.R.); 5 Department of Bioproducts and Biosystems, Aalto University, 00076 Espoo, Finland; ahmed.1.ahmed@aalto.fi (A.S.); jinze.dou@aalto.fi (J.D.)

**Keywords:** menadione, picein, ROS, MitoSOX, live cell imaging, oxidative stress, neuroblastoma SH-SY5Y cells, neurodegenerative disorder

## Abstract

Several bioactive compounds are in use for the treatment of neurodegenerative disorders, such as Alzheimer’s and Parkinson’s disease. Historically, willow (*salix* sp.) bark has been an important source of salisylic acid and other natural compounds with anti-inflammatory, antipyretic and analgesic properties. Among these, picein isolated from hot water extract of willow bark, has been found to act as a natural secondary metabolite antioxidant. The aim of this study was to investigate the unrevealed pharmacological action of picein. In silico studies were utilized to direct the investigation towards the neuroprotection abilities of picein. Our in vitro studies demonstrate the neuroprotective properties of picein by blocking the oxidative stress effects, induced by free radical generator 2-methyl-1,4-naphthoquinone (menadione, MQ), in neuroblastoma SH-SY5Y cells. Several oxidative stress-related parameters were evaluated to measure the protection for mitochondrial integrity, such as mitochondrial superoxide production, mitochondrial activity (MTT), reactive oxygen species (ROS) and live-cell imaging. A significant increase in the ROS level and mitochondrial superoxide production were measured after MQ treatment, however, a subsequent treatment with picein was able to mitigate this effect by decreasing their levels. Additionally, the mitochondrial activity was significantly decreased by MQ exposure, but a follow-up treatment with picein recovered the normal metabolic activity. In conclusion, the presented results demonstrate that picein can significantly reduce the level of MQ-induced oxidative stress on mitochondria, and thereby plays a role as a potent neuroprotectant.

## 1. Introduction

**Figure 1:**
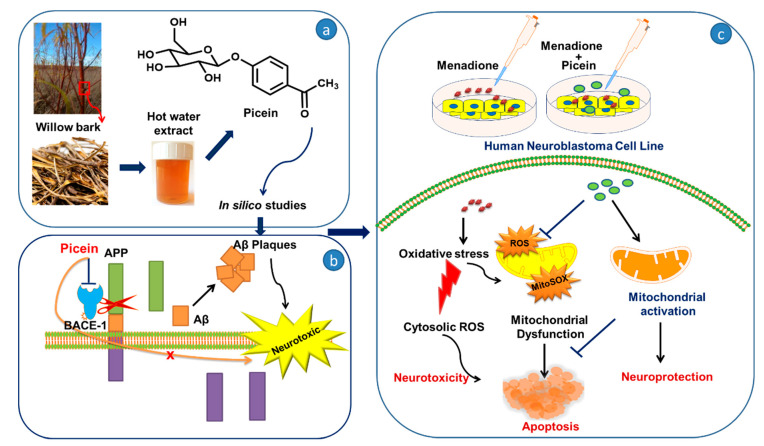
An overview of the experimental plan representing (**a**) the isolation of picein from willow bark [18] followed by its (**b**) in silico and (**c**) in vitro studies. In silico studies for the identification of putative biomolecular target reveals Beta Secretase 1 (BACE-1) as the highest-ranked target enzyme for picein, which might indicate its neuroprotective role. Amyloid precursor protein (APP) is a prerequisite for β-amyloid (Aβ) formation, which is sequentially cleaved by BACE1. In vitro studies on SHSY5Y cells shows that menadione induces mitochondrial ROS, superoxide production and also mitochondrial depolarization which lead to mitochondrial damage and ultimately apoptosis of the cell. Furthermore, subsequent treatment with picein recovers the mitochondrial function through its antioxidative effects and thus plays an important role in neuroprotection.

In recent years, the growing percentage of neurodegenerative diseases has been attributed to an induced oxidative stress array of human health, environment, and lifestyle factors [1,2]. It is known that the physiology of neurodegenerative diseases is multifactorial, and several recent studies confirm that potential superficial factors like radiation [3,4] or chemical compound exposures [5,6,7] are associated with the risk of the onset of neuronal diseases. Exogenous compounds such as menadione (MQ) (2-methyl-1,4-naphthoquinone), offer an examination of the oxidative stress-induced cytotoxicity in the cell, where due to the formation of reactive oxygen species (ROS), the cell either becomes dysfunctional or dies (Figure 1) [8,9]. Such a situation may occur if the ROS formation exceeds the scavenging capacity of the antioxidants. The cytotoxic effect of menadione-induced oxidative stress may occur in distinct redox states and its cycling may generate ROS in target cells [10], which may cause cell injury and result in the pathogenesis of various neurodegenerative disorders, for example Parkinson’s disease (PD) [11] and most commonly Alzheimer’s disease (AD) [12,13,14,15]. According to the World Alzheimer report, 46.8 million people worldwide are living with dementia in 2015 [16]. Alarmingly, this forecasted number could double or triple after every 20 years through to the year 2040, which would lead to an expensive burden of disease on society in the future [16,17].

Continuing efforts through the years have been made to find an antioxidant that can lower the accelerating aging of neurons due to oxidative damage leading to neurodegenerative diseases, also reported by Poljsak and Milisav [19]. Therefore, this study aimed to explore the neuroblastoma SH-SY5Y cell type-specific responses to menadione-induced oxidative stress and repair mechanisms by using the authentic compound ‘picein’ that is also present in willow bark water extracts [16]. Willow bark extracts are known as a rich source of antioxidants and have significant potential for medical applications [20,21,22]. Willow bark has shown importance in the past with the discovery of aspirin (acetylsalicylic acid) for the treatment of anti-inflammatory, antipyretic and analgesic conditions [23]. Recently from our group, Dou et al. [18] demonstrated a cost-effective and efficient method for extracting the bioactive compounds picein, triandrin and catechin from the bark of *salix* hybrid Karin. In this study, a bioinformatics approach was first applied on extracted compounds reported by Dou et al. [18] and thereafter picein was opted for use in in vitro studies due to its strong role in the treatment of AD. In vitro studies were performed on SH-SY5Y neuroblastoma cells because of their close resemblance to neurons and they are accepted as a unique model for neurodegenerative diseases [24,25,26].

For the measurements of oxidative stress, we examined the cellular changes induced by MQ (highly responsible for causing DNA damage and ROS production) at several concentrations followed by the neuroprotectant picein (antioxidant) in menadione-treated neuroblastoma cells. To confirm the role of menadione and picein, live-cell imaging was assessed as a measure of mitochondrial dysfunction. Mitochondrial superoxide and ROS production were measured as indicators of oxidative stress along with mitochondrial activity using thiazolyl blue tetrazolium bromide (MTT). The rationale for measuring these endpoints was to establish a link between neurodegeneration and AD. Although, several researchers have reported that oxidative stress-induced ROS formation may be the link to human health, and leads to pathological diseases, which has been found as a leading cause of death [27,28,29].

Our hypothesis is based on the qualitative and functional similarity of picein (as one of the Karin bark water extract’s component), to that of the bioactive compound, ‘gastrodin’ extracted from the dried rhizome of *Gastrodia elata*. Gastrodin is a well-known phenolic glycoside, which possess neuropharmacological properties such as anti-inflammatory, antioxidative, and modulating the secretion of neurotransmitters that suppresses the activation of microglia and regulates the mitochondrial cascade reactions like mitochondrial durability and functionality [30]. Many researchers have reported a significant role of gastrodin in neurological diseases such as AD [31,32,33], PD [34,35,36], and depression [37,38]. Structurally, picein showed a 92% similarity score with gastrodin. In this consideration, we performed in silico and in vitro studies to explore the scavenging capacity of picein against menadione. This is the first report to our knowledge showing the potential role of picein as neuroprotectant which attenuates the cytotoxic effect of menadione-induced oxidative stress in neurodegenerative diseases.

## 2. Materials and Methods

### 2.1. Chemical Similarity Search

SIMCOMP (SIMilar COMPound), a plug-in of the Kyoto Encyclopedia of Genes and Genomes (KEGG) database, was used for the chemical structure similarity search. This software was used for the selection and identification of similar or identical chemical structures to predict the analog of the query compound on the basis of a graph-based method [39]. Similarity search is an important parameter to reach any certain prediction as we can see in the present study of picein and gastrodin [39].

### 2.2. Testing of Lipinski’s Rule of 5, Generation of 3D Model of Protein and Ligands and Docking Studies

The chemical descriptor and drug-like properties of picein (PubChem ID: 92123) and gastrodin (PubChem ID: 115067) were observed by using Lipinski’s rule of 5 (www.molinspiration.com), where this rule was specially designated for the selection of small druggable compounds [40,41]. Conical SMILE IDs is a specific and particular chemical structure characteristic of every chemical compound which is usually mentioned in chemical databases. PubChem database, CORINA 3D server is an important software which converts the SMILE ID into a three-dimensional *.pdb* structure. The RCSB protein data bank (Research Collaboratory for Structural Bioinformatics protein data bank) was used for procuring the 3D structure of amyloid beta-peptide (PDB ID: 1IYT). Auto Dock Tool 4 (MGL Tool: Molecular graphics laboratory) is a globally accepted docking tool, which was used in this study to identify the poses of ligands, proteins and the binding affinities [42].

### 2.3. Identification of Putative Biomolecular Target and Protein-Protein Interaction Analysis

A reverse docking (PharmMapper Server) approach was used to identify the putative targets of picein and gastrodin. This web-server identifies the most probable target candidates of small-scale molecules on the basis of a pharmacophore mapping approach. Protein-protein interactions and topological analysis were performed using a CHIPPI (CHimeric protein-protein interaction server) web-server [43].

### 2.4. Mammalian Cell Culture

For the experiments, human SH-SY5Y neuroblastoma cells (collected from Dr. Juan Cruz Landoni, Research Program for Molecular Neurology, University of Helsinki) were obtained and grown in Dulbecco’s modified Eaglés medium (DMEM). For cell culture, 10% foetal bovine serum (FBS) along with a mixture of streptomycin (50 μg/mL)/penicillin (50 U/mL) was added in DMEM (4.5 g/L glucose). The SH-SY5Y cell cultures were grown and maintained in Eppendorf cell culture flasks (T-75, Catalogue 0030711025) at 37 °C in a humidified incubator with supply of 5% CO_2_. For experimental assays, cells were trypsinized by 0.2% trypsin and seeded at a density of 2 × 10^5^ cells in 48-well plates (Costar, Corning, NY, USA).

### 2.5. Experimental Design for In Vitro Studies

Experiments were conducted to confirm the neuroprotectant role of picein in a changing level of mitochondrial superoxide and ROS production by suppressing the effect of MQ. Menadione was used to induce oxidative stress and cause ROS formation in the cells. Picein (74192) and menadione (MQ) (M5625) were purchased from Sigma Aldrich, USA. For the experiments, the following treatment groups were taken—(1) control, (2) Picein (3) MQ, (4) MQ + Picein. Induction of oxidative stress involves the incubation of cells with MQ for 2 h with the subsequent treatment with picein for 2.5 h. The MQ concentrations were 0, 1, 10, 15 and 20 μM and the picein concentration was fixed at 25 μM. Dose-response experiments were employed for the standardization of picein concentration, where concentrations of 1, 10, 15, 25, 50, and 100 μM were used to obtain the higher cell survival rate (data not presented). We obtained the best cell survival rate at 25 μM of picein, although 20 μM was the maximum dose for menadione recorded. Commercially available authentic picein (≥98.0% HPLC grade) was used for the treatment of menadione-stressed neuroblastoma cells during the present study. Live cell imaging, ROS, mitochondrial superoxide, and mitochondrial activity (MTT), were performed immediately after the picein treatment. A blank (without cells) was involved in the assay for the measurements, where the absolute values were subtracted from the blank value.

### 2.6. Mitochondrial Activity (MTT) Assay

Mitochondrial activity was measured using 3-(4,5-dimethylthiazol-2-thiazolyl)-2,5 diphenyl tetrazolium bromide or thiazolyl blue tetrazolium bromide (MTT; Sigma Aldrich, Saint Louis, MO 63103, USA). Immediately after the menadione and picein treatment, the medium was removed from the 48-well plates and replaced with the assay-specific probe in 250 μL complete media containing 25 μL MTT solution (5 mg/mL) and kept for 3 h of incubation in the incubator. After incubation to dissolve formazan crystals, MTT solution was replaced with 250 μL DMSO in each well. The reading of absorbance was taken with a microplate reader at a wavelength of 550 nm (Cytation 3, BioTek Instruments, Inc., Winooski, VT, USA).

### 2.7. Reactive Oxygen Species (ROS) Formation

ROS production was assayed using 2′,7′-dichlorofluorescein diacetate (DCFH-DA) from a Sigma-Aldrich (St. Louis, MO, USA) assay-specific probe. Immediately after the menadione and picein treatment, the medium was removed from the 48-well plates and replaced with the assay-specific probe DCF-DA (40 μM) in 0.5 mL of Hank’s balanced salt solution and thereafter incubated for 30 min in the incubator. Further, fluorescence was measured at 485 nm excitation/535 nm emission wavelengths by a Biotek Cytation Reader 3 (BioTek Instruments, Inc., Winooski, VT, USA).

### 2.8. Mitochondrial Superoxide Production

Mitochondrial superoxide production was analyzed as described previously [44]. A fluorescent probe, MitoSOX red (3,8-phenanthridinediamine, 5-(6′-triphenylphosphoniumhexyl)-5,6-dihydro-6-phenyl) (M36008) was purchased from Molecular Probes (Thermo Fisher Scientific, 29851 Willow Creek Road, Eugene, OR 97402, USA). Briefly, for the analysis of mitochondrial superoxide production, MitoSOX red with a final concentration of 4 µM was added in phosphate buffer saline (PBS). Immediately after picein treatment, the medium was removed and washed with PBS and thereafter, the assay-specific probe in 0.5 mL of buffer for 15 min incubated in the incubator at 37 °C. After incubation, the probe was replaced with warm buffer, and fluorescence intensity was measured at 510 nm excitation/640 nm emission wavelengths by a Biotek Cytation Reader 3 (BioTek Instruments, Inc., Winooski, VT, USA).

### 2.9. Live Cell Imaging

The SH-SY5Y neuroblastoma cells were allowed to attach at the glass-bottom well plates by following all the treatment processes. Thereafter, the cells were washed thrice with serum-free DMEM and 0.2 uL of Mitotracker orange (MitoTracker^®^, Thermo Fisher Scientific, Eugene, OR, USA) was added first to 1.5 mL of medium and then poured drop by drop to the dish. Cells were incubated at 37 °C with a supply of 5% CO_2_ for 30 min and further maintained at similar conditions during the imaging. The live-cell imaging was performed with a confocal microscope (Leica TCS SP5, Leica Microsystems, Am Friedensplatz 3. 68165 Mannheim, Germany) and images were obtained by using a 63X water objective. The Mitotracker orange was excited by a 561 nm diode laser. The fluorescence signal was collected at 575–610 nm with PMTs (Photomultiplier tubes) for Mitotracker orange.

### 2.10. Statistical Analysis

For the statistical analysis, a general liner model procedure of SPSS was carried out to see the statistically significant differences between groups. In this process, factorial ANOVA (in factorial design) was applied, where MQ and picein were allocated as fixed factors and in the same model replicate as random factors. Also, to check the significant differences within groups, a paired t-test was applied. A difference in *p*-value less than 0.05 was reported statistically significant.

## 3. Results

### 3.1. Chemical Similarity Search

SIMCOMP (SIMilar COMPound), a plug-in of KEGG database system, was used for the identification of similar compound properties like picein. On the basis of a graph-based method of this database, gastrodin was found to have a similarity score of 0.92 (gastrodin shares 92% chemical structure similarity with Picein) out of 1, and spotted to have the highest analogy or similarity with picein (Table 1). This shows that picein and gastrodin are similar in chemical structure, and this supports the characteristics of bioactive compounds for disease treatment.

### 3.2. Identification of Putative Biomolecular Target and Protein-Protein Interaction

Based on the pharmacophore mapping, PharmMapper web-server was used to predict the putative biomolecular targets of picein (Job no. 180512174402, no. of feature: 18, fit score: 3.557, normalized fit score: 0.1976, Z-Score: 0.345543) and gastrodin (Job no. 180512174552, no. of feature: 18, fit score: 3.883, normalized fit score: 0.2157, Z-Score: 0.896571). After screening both of compounds, Beta Secretase 1 (BACE-1) was found to be the best-ranked target for both of the query compounds.

**Figure 2:**
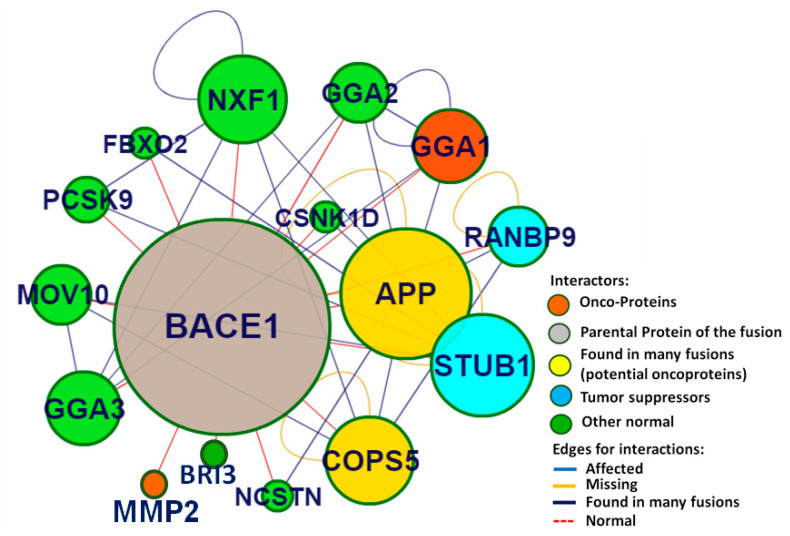
CHIPPI web server was used for the network protein-protein interaction (PPI) analysis. This study advocated that BACE1 can be a preferential drugable target. According to topological analysis, APP (amyloid-beta precursor protein) was found to be the major important neighboring protein of BACE1.

CHIPPI was used for the network protein-protein interaction (PPI) analysis. In this study, BACE1 was found to be a perfect drug target. The PPI size of BACE1 was found to be 15, which shows that in this network 15 different proteins are associated with the clustering coefficient, and the betweenness centrality of BACE1 was 0.19 and 128.333, respectively. The topological analysis suggests that the amyloid-beta precursor protein (APP) was the major neighboring protein of BACE1 (Figure 2), and its potential role in the pathogenies of neurodegenerative diseases has also been reported.

### 3.3. Testing of Lipinski’s Rule of 5 and Docking Analysis

**Figure 3:**
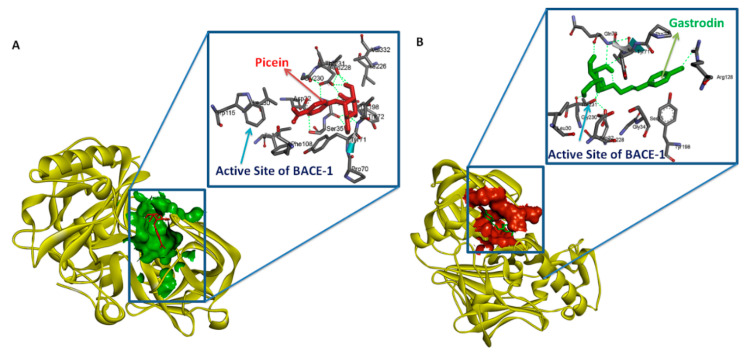
Binding interaction pattern and poses of gastrodin with BACE1 (**A**) and picein with BACE1 (**B**), where picein (−5.94 Kcal/Mol) is exhibiting stronger binding affinity than gastrodin (−5.78 Kcal/Mol). Both of ligands share almost the same binding site.

This study was focused on identifying the chemical descriptors along with druglike and molecular binding properties of picein and gastrodin. The notation for picein and gastrodin were LogP −0.43 and −0.99, topological polar surface area 116.45 and 119.61, molecular weight 298.29 and 286.28 gmol^−1^, the total number of hydrogen bond acceptors seven and seven, hydrogen donors five and four, and the total number of rotatable bonds were four and four, respectively. Thus, neither picein nor gastrodin has been observed to violate any rule of Lipinski’s. The binding affinities and *K*_i_ value (dissociation constant) of BACE1 with picein were −5.94 Kcal/Mol and 44.03 µM. Similarly, the binding affinity and *K*_i_ values of gastrodin with BACE1 were −5.78 Kcal/Mol and 57.49 µM (Table 2). The molecular interaction pattern of both compounds with BACE1 is shown in Figure 3.

### 3.4. Mitochondrial Activity (MTT)

**Figure 4:**
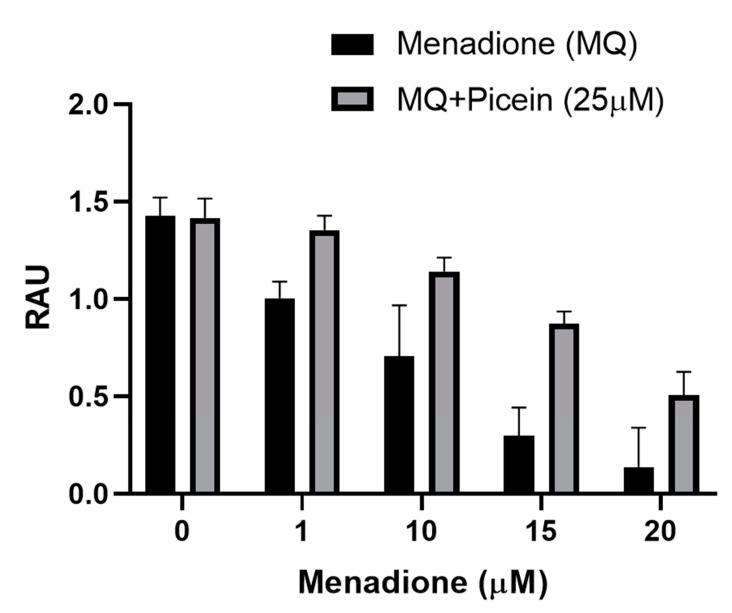
Level of mitochondrial activity (MTT) in human SH-SY5Y neuroblastoma cells after menadione (MQ) treatment and follow-up with picein. The data are presented as mean ± SEM. To give evidence for the statistical variations, factorial ANOVA was applied to examine the *p*-values for each factor, where three experiments with three samples per group were considered. Significant differences were seen in picein (*p* = 0.002), and MQ (*p* < 0.0001). RAU indicates relative absorbance units.

The mitochondrial activity was measured to confirm the effect of picein in menadione-exposed SH-SY5Y cells, where cells were exposed first with menadione (concentrations—1, 10, 15 and 20 µM), followed by the treatment with a fixed concentration of picein (25 µM). Cells individually treated with menadione resulted in a statistically significant lower cell viability when compared to the control cells (Figure 4). Subsequently, follow-up treatment with picein significantly inhibited the effect of menadione and resulted in a recovery of damaged cells with an increasing concentration of menadione (MQ). Individually picein-treated SH-SY5Y cells (without MQ), did not show any changes in the level and was close to the control group.

### 3.5. Reactive Oxygen Species

**Figure 5:**
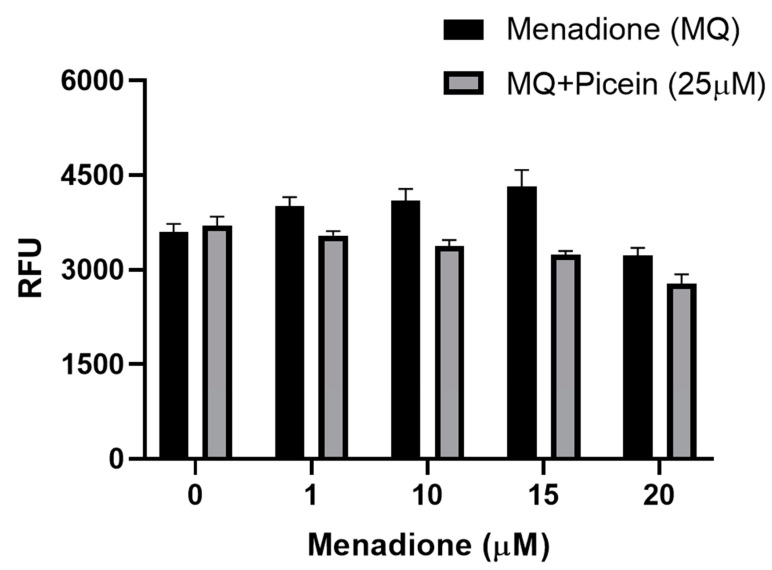
Reactive oxygen species level in human SH-SY5Y neuroblastoma cells were measured after menadione (MQ) treatment and follow-up treatment with picein. The data are presented as mean ± SEM. To give evidence for the statistical variations, factorial ANOVA was applied to examine the *p*-values for each factor, where three experiments with three samples per group were considered. Statistically significant differences were examined for picein (*p* < 0.0001) and MQ (*p* < 0.0001). RFU indicates relative fluorescence units.

For the measurements of ROS formation, SH-SY5Y cells were first exposed with menadione (concentrations—1, 10, 15 and 20 µM), followed by the picein treatment (25 µM). Cells individually treated with menadione (without picein) resulted in a statistically significant increase in the ROS level than in the corresponding control group (Figure 5), although a decrease was detected at 20 µM MQ. Afterwards, a significant decrease in the level of ROS was detected in the follow-up treatment with picein. These results show that picein inhibited the effect of MQ by reducing ROS formation. No significant changes were seen in SH-SY5Y cells individually treated with picein (without MQ).

### 3.6. Mitochondrial Superoxide Production

**Figure 6:**
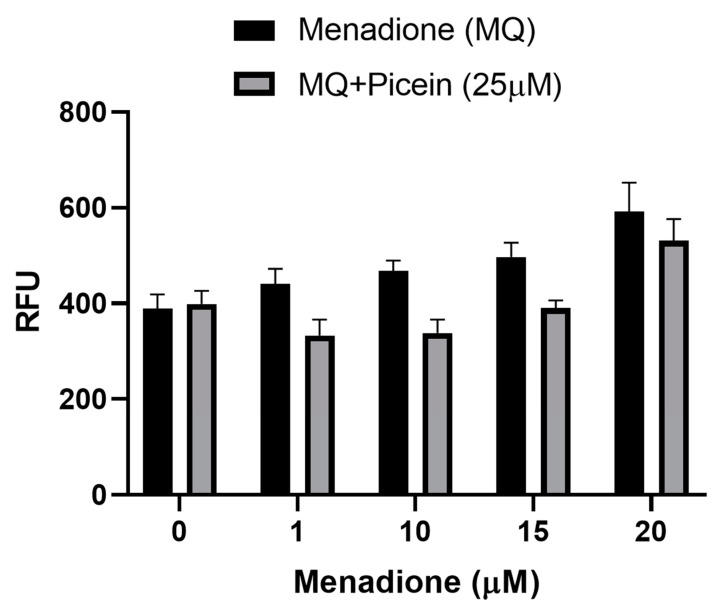
Level of mitochondrial superoxide in human SH-SY5Y neuroblastoma cells were measured after menadione (MQ) treatment and follow-up treatment with picein. The data are presented as mean ± SEM. To give evidence for the statistical variations, factorial ANOVA was applied to examine the *p*-values for each factor, where three experiments with three samples per group were considered. Statistically significant differences were examined for picein (*p* < 0.0001), and MQ (*p* < 0.0001). RFU indicates relative fluorescence units.

The antioxidative effect of picein in menadione-exposed SH-SY5Y cells was also examined to observe the mitochondrial superoxide production, where cells were exposed first with menadione (concentrations—1, 10, 15 and 20 µM), followed by the treatment with picein (25 µM). A statistically significant increase in the mitochondrial superoxide level (particularly at 20 µM MQ concentration) was measured in the cells individually treated with menadione (without picein) in corresponding concentrations to the control group (Figure 6). Subsequently, a significant decline in the mitochondrial superoxide level was also observed in the follow-up treatment with picein, showing the damaged cell retrieval effect with increasing concentrations of menadione (MQ). No significant changes were seen in SH-SY5Y cells individually treated with picein (without MQ).

### 3.7. Live Cell Imaging

**Figure 7:**
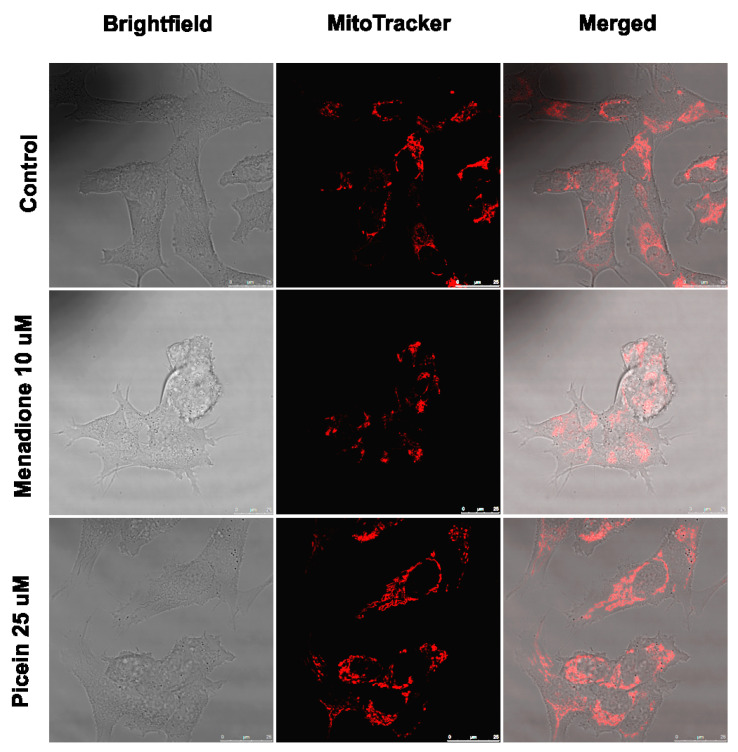
Live cell microscopy of human SH-SY5Y neuroblastoma cells immediately after menadione (MQ) exposure and follow-up treatment with picein. All images were taken with a 63X water objective. Images were taken in brightfield and Mitotraker mode. Further merged these images to see the morphological differences among control, menadione (10 µM) and follow-up treatment with picein (25 µM).

The live cell imaging of SH-SY5Y neuroblastoma cells was performed to visually verify the neuroprotective effects of picein. Microscopy with Mitotracker orange (stains mitochondria in live cells) was performed with control, menadione-treated (10 µM) and picein-treated (25 µM) samples after menadione exposure. The live cell imaging of SH-SY5Y cells after menadione exposure showed low emission of Mitotracker fluorescence, could be indicating an overproduction of ROS, as also reported by Tomkova et al. [45]. Moreover, the qualitative observations from Figure 7 clearly reveal that the menadione-exposed cells were morphologically distinct with structural abnormalities, and such deviations could be interpreted as mitochondrial dysfunction, as has been reported by other researchers [46,47]. However, the follow-up treatment with picein of menadione-stressed cells showed an increase in fluorescence intensity of Mitotracker, suggesting a decrease in ROS production with an increase in mitochondrial membrane potential (Figure 7). Consequently, these results showed that picein may lead to a significant recovery of damaged cells with a comparable morphology similar to that of untreated control cells.

## 4. Discussion

In this study, human SH-SY5Y neuroblastoma cells were used to study the oxidative stress responses of menadione with the subsequent neuroprotective effect of picein. In addition to live cell imaging, ROS and mitochondrial superoxide production were observed along with mitochondrial activity, as these parameters are consistent with the suspected adverse response to menadione by human neuroblastoma cells. Thus, this study aimed to monitor the oxidative stress levels in SH-SY5Y cells and further the stress inhibitory role of picein in a follow-up treatment. The hypothetical concept of the present study was developed from our recent publication Dou et al. [18], where picein was extracted from willow bark (*Salix* sp.) and further medicinal applications were analyzed by a computational approach. Picein is a mispriced and cryptic compound in the scientific domain, on which any neurological study has not yet been published to the best of our knowledge. Therefore, this study explores the basic chemical (in silico) and biological properties of picein to identify its putative role in the list of natural and bioactive compounds.

Primarily, the chemical structure similarity search was performed to identify the closest analogue of picein and to predict its putative biological activity. Gastrodin is the closest analogue of picein, for which a 0.92 similarity score out of 1 was obtained after a computational approach (Table 1). Gastrodin has been identified as a natural bioactive compound of *Rhizoma Gastrodia*, a dried rhizome of *Gastrodia elata Blume (G. elata)*, tested as a popular Chinese herb for the treatment of epilepsy, stoke, spasm, and dizziness and headache [48]. The studies of Feng et al. [49] and Zhou et al. [50] have reported that the gastrodin isolated from *G. elata* has promising pharmacological properties. Since then, this compound has been recognized as a proven sedative, hypnotic, antivertigo, antiepileptic, antidepressant, anxiolytic, and memory-improving agent [30]. Several other studies also reported that gastrodin (5000 research articles) is actively being investigated for its role in the treatment of neurological diseases [31,32,33], Alzheimer’s disease [34,35,36] and depression [37,38].

Based on the similarity scores and pharmacological properties of gastrodin [51], picein is considered as a neuroprotective compound. Additionally, Lipinski’s rule of five was also analyzed from a computational approach to affirm the drug-likeness of both compounds regarding their antioxidative capacity (Figure 3). Thus, this study mainly focusses on the role of picein in neuroprotection. From here, this study led toward targeting the identification step, where the PharmMapper web-server was used to screen out the probable biomolecular targets of both compounds. Interestingly, by the reverse docking approach, beta-secretase 1 (BACE-1) was found as the best biomolecular target for both compounds (Figure 4). These results confirm the chemical and biological similarity between both of the query compounds. In beta-secretase 1, the beta-site APP-cleaving enzyme is an aspartic-acid protease, which has great importance for the myelin sheath development in peripheral nerve cells [52]. However, its over-activation may lead to the genesis and cleavage of APP (amyloid-beta precursor protein), which has been recognized as a key biomarker for the progression of neurodegenerative diseases like AD [52]. For the cross-validation of the relation between BACE1 and APP, we have performed the network topological and protein-protein interaction analysis and found the strong relationship between BACE-1 and APP (Figure 3). Network topological analysis shows that the clustering coefficient of BACE1 was 0.19. However, if the clustering coefficient (Pearson correlation coefficient) is found to be less than 0.5, it could be simulated to bind a distinct ally at particular time points and restricted to date hubs. These date proteins are important in PIN, but hold a lower number of connections. Therefore, chances of the binding of drug or other ligands would be higher than party proteins (party proteins, co-efficient more than 0.5 and show a high degree of co-expression with interaction nodes/partner were assumed to interact at the same time with their interaction nodes/partners) [53]. However, betweenness centrality was 128.33, and higher betweenness controls most of the data flow in the network, which may represent the critical points of the network [54]. Hence, these topological parameters strongly support BACE1 as a suitable drug target of picein and gastrodin (Figure 3). Molecular docking studies also helped us to find out the preferential binding capability of both compounds with BACE1, where picein has found better binding ability towards BACE1 than gastrodin. The biocomputational results narrate our hypothesis that picein behaves as a better neuroprotectant than gastrodin.

For the implementation of biocomputational findings, cell culture-related endpoints were performed to justify the proposed hypothesis and mechanism of picein for the treatment of neurodegenerative diseases. The experiments were directed to confirm the effects of picein, and the expected responses were detected, where a decreased ROS level was measured in the picein-treated cells compared to the menadione-treated cells (Figure 1). Moreover, treatment with picein blocked the effect of menadione and resulting in a decreased ROS level close to the control cells. A systematically increasing trend in the ROS level was observed after individual treatment of menadione, where it was higher at 1, 10 and 15 µM and decreased at a 20 µM concentration (Figure 5). The decrease in the ROS level at higher concentrations was obvious, due to lower and under-stressed cell conditions. Similarly, Kang et al. [55] also investigated the enhanced level of ROS after short menadione exposures on neuroblastoma cells, while longer treatment resulted in a lowering of the ROS level. This was also confirmed by mitochondrial activity, where cells were stressed with increasing concentrations of menadione. Picein at a fixed concentration of 25 μM significantly blocked the menadione effects on cells by inhibiting the cells with oxidative damage (Figure 4). MTT is a well-known assay for the detection of compounds that interfere with or produce alterations in mitochondrial activity [56]. However, in living cells, mitochondria turn yellow MTT to purple formazan, and this could be because of live cells reacting with tetrazolium salts as active mitochondrial dehydrogenase enzymes, cleaving the tetrazolium ring [57,58].

Menadione produces intracellular ROS over futile redox cycling at multiple cellular sites, which also depends on the concentrations. Consequently, menadione-conciliated oxidant responses at lower concentrations may activate redox-dependent gene expression [59]. However, menadione-induced oxidative stress at higher concentrations is linked with the cellular injury and cell death [59,60,61,62]. The present study is in agreement with our previous work, where Kesari et al. [44,63] also reported that the menadione-induced oxidative stress increases the ROS level in addition to mitochondrial superoxide production in SH-SY5Y cells. Thus, the mitochondria-specific probe MitoSOX Red was employed to detect the superoxide production and to assess the integrity of mitochondria [64,65]. Several recent studies reported that the ROS formation in mitochondria may lead to mitochondrial superoxide production [44,66,67]. In this study, menadione treatment increases mitochondrial superoxide production after exposure to the increasing trend (Figure 6). Thereafter, treatment with picein was found to block the menadione effect and the level of superoxide was kept close to the control cells. MitoSOX red, a novel fluorogenic probe, can bind to the nuclear DNA and therefore identify superoxide in the live cells of mitochondria [64]. In addition to this, the selective mitochondrial probe Mitotracker orange was employed in this study, which is highly receptive to mitochondrial membrane potential and considered a sensitive indicator for the measurement of oxidative stress in mitochondria [45]. The accumulation of Mitotracker in cells mainly depends on the mitochondrial membrane potential [68] because it disseminates throughout the plasma membrane and accumulates in live mitochondria. Figure 7 shows a decreased Mitotracker fluorescence intensity in the menadione-treated group as compared to control and picein-treated groups. Lower Mitotracker fluorescence results in ROS production and could be acknowledged as the reliable assay to examine the oxidative stress even at low-level ROS formed in mitochondria. Kweon et al. [69] reported that the intracellular superoxide production can be detected by Mitotracker at the early phase and later may be converted to H_2_O_2_. The proposed mechanism of picein for blocking the effect of menadione has been presented in Figure 1. The data of this study, therefore, highlight the importance of advances in drug discovery methods by undertaking several highly specified molecular biology and computational parameters.

## 5. Conclusions

The findings of the present study mainly conclude the statistically significant decrease in ROS levels and superoxide production after treatment with picein, indicating its neuroprotective properties. Additionally, mitochondrial activity (MTT) showed significant recovery of cells after picein treatment, which indicates the inhibition of mitochondrial ROS formation. Moreover, live cell imaging was employed as an indicator of mitochondrial oxidative stress, where low absorption of Mitotracker fluorescence indicated an overproduction of ROS. In silico modeling of the molecular structure of picein resulted in a surprising similarity of 92% with gastrodin. Although gastrodin is well-known for its role in neuroprotection, picein was found to possess better neuroprotective properties than gastrodin. Picein as a neuroprotectant has great possibilities towards drug formulation first with animal model experiments. However, based on the present study and in vitro model, picein could be used in transgenic APP/PS1 mice (AD animal model) for the treatment of neurodegenerative diseases for future research. We have also identified a novel BACE1 inhibitor, picein, which acts as a strong neuroprotectant and has therapeutic potential for AD treatment. The overview of the whole study is presented in Figure 1.

Consequently, the present study confirms the antioxidative property of picein against the oxidative stress induced by menadione in human neuroblastoma cells. Therefore, this study lays a great foundation to further explore the development of drugs for neurodegenerative diseases based on picein, along with the other extracted compounds of a single clone willow hybrid Karin. The extract composition of compounds varies greatly between the hundreds of individual willow hybrids that have been developed so far. So, in the future, there are great possibilities to progress in different areas of research where the biological activity of the extracts is important.

## Figures and Tables

**Table 1:** SIMCOMP (SIMilar COMPound) similarity score of picein and gastrodin.

S. No.	Chemical Name	Chemical Structure	Similarity Score
1	Picein	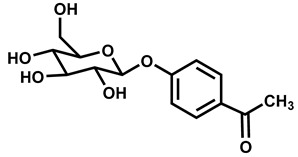	1.0
2	Gastrodin	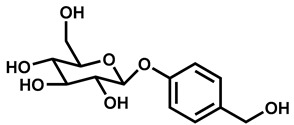	0.92

**Table 2:** Binding interaction analysis of gastrodin and picein with BACE1.

S. No.	Rece-Ptor	Ligand	Binding Energy Kcal/Mol	*K*i	Hydrophobic Interaction	Hydrophilic Interaction	Length of H Bond (Ǻ)
1	BACE1	Gastrodin	−5.78 Kcal/Mol	57.49 µM	Leu30, Asp32, Gly34, Ser35, Gln73, Pro70, Tyr71, Thr72, Arg128, Tyr198, Asp228, Thr231, Gly230	BACE1:THR72:N-Gastrodin:O20	2.93955
						BACE1:GLN73:N-Gastrodin:O20	3.03446
						BACE1:ARG128:NH2-Gastrodin:O1	2.77309
						BACE1:THR231:OG1-Gastrodin:O22	2.91867
						Gastrodin:H42-BACE1:THR231:OG1	2.23271
						Gastrodin:H36-BACE1:GLY230:O	2.18995
						Gastrodin:H38-BACE1:GLN73:O	1.8119
						BACE1:THR72:N-Gastrodin:O20	2.93955
						BACE1:GLN73:N-Gastrodin:O20	2.77309
						BACE1:ARG128:NH2-Gastrodin:O1	3.03446
						BACE1:THR231:OG1-Gastrodin:O22	2.91867
						Gastrodin:H42-BACE1:THR231:OG1	2.23271
						Gastrodin:H36-BACE1:GLY230:O	1.8119
						Gastrodin:H38-BACE1:GLN73:O	2.18995
2	BACE1	Picein	−5.94 Kcal/Mol	44.03 µM	Ser35, Leu30, Asp32, Gly34, Pro70, Thr72, Phe108, Trp115, Tyr71, Ile118, Tyr198, Thr231, Ile226, Asp228, Gly230, Val332	BACE1:THR72:N-Picein:O12	3.03172
						BACE1:THR231:OG1-Picein:O21	2.55481
						Picein:H39-BACE1:ASP228:OD2	2.20644
						Picein:H39-BACE1:THR231:OG1	1.96118
						Picein:H37-BACE1:ASP228:OD2	1.74178
						Picein:H33-BACE1:GLY34:O	2.24183
						Picein:H35-BACE1:GLY34:O	1.87567

## Abbreviations

**AD**	Alzheimer disease
**Aβ**	Amyloid beta
**APP**	Amyloid-beta precursor protein
**BACE-1**	Beta secretase enzyme
**CHIPPI**	CHimeric protein-protein interaction server
**DNA**	Deoxyribonucleic acid
**DMEM**	Dulbecco’s modified Eaglés medium
**DMSO**	Dimethyl sulfoxide
**DCFH-DA**	2′,7′-dichlorofluorescein diacetate
**PBS**	Phosphate buffer saline
**FBS**	Foetal bovine serum
**HPLC**	High performance liquid chromatography
**KEGG**	Kyoto encyclopedia of genes and genomes
**MTT**	Thiazolyl blue tetrazolium bromide
**MQ**	Menadione
**MGL Tool**	Molecular graphics laboratory tool
**MitoSOX**	Mitochondrial superoxide
**PD**	Parkinson disease
**PPI**	Protein-protein interaction
**RFU**	Relative fluorescence units
**RAU**	Relative absorbance units
**RCSB**	Research collaboratory for structural bioinformatics protein data bank
**ROS**	Reactive oxygen species
**SH-SY5Y**	Neuroblastoma cell
**SIMCOMP**	SIMilar COMPound.
